# UV‐Induced Charge‐Transfer States in Short Guanosine‐Containing DNA Oligonucleotides

**DOI:** 10.1002/cbic.202000103

**Published:** 2020-05-05

**Authors:** Corinna L. Kufner, Wolfgang Zinth, Dominik B. Bucher

**Affiliations:** ^1^ Biomolecular Optics and Center for Integrated Protein Science Ludwig-Maximilians-University Munich Oettingenstr. 67 80538 Munich Germany; ^2^ present affiliation: Harvard-Smithsonian Center for Astrophysics Department of Astronomy Harvard University 60 Garden Street Cambridge MA 02138 USA; ^3^ present affiliation: Department of Chemistry Technical University of Munich Lichtenbergstr. 4 85748 Munich Germany

**Keywords:** charge transfer, DNA damage, guanine, photophysics, picosecond IR spectroscopy

## Abstract

Charge transfer has proven to be an important mechanism in DNA photochemistry. In particular, guanine (dG) plays a major role as an electron donor, but the photophysical dynamics of dG‐containing charge‐transfer states have not been extensively investigated so far. Here, we use UV pump (266 nm) and picosecond IR probe (∼5–7 μm) spectroscopy to study ultrafast dynamics in dG‐containing short oligonucleotides as a function of sequence and length. For the pure purine oligomers, we observed lifetimes for the charge‐transfer states of the order of several hundreds of picoseconds, regardless of the oligonucleotide length. In contrast, pyrimidine‐containing dinucleotides d(GT) and d(GC) show much faster relaxation dynamics in the 10 to 30 ps range. In all studied nucleotides, the charge‐transfer states are formed with an efficiency of the order of ∼50 %. These photophysical characteristics will lead to an improved understanding of DNA damage and repair processes.

## Graphical abstract

**Studying the photophysical dynamics** of dG‐containing oligonucleotides by time‐resolved IR spectroscopy showed transient absorption changes pointing to excitation decay and vibrational cooling within a few picoseconds. On the 10‐500 picosecond time range transient charge‐transfer states were identified which are efficiently formed in these oligomers. The known reactivity and the high formation yield make charge‐transfer states important promoters for secondary reactions in DNA.

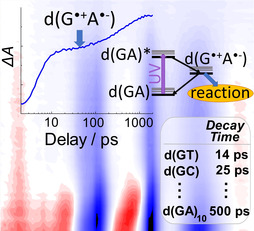

## Introduction

UV irradiation induces a selection pressure to which DNA molecules have been exposed for several billions of years on the surface of the earth.^1^ It is assumed that the selection of the four canonical DNA mononucleotides is related to photostability provided by the ultrashort lifetimes of the excited states.^2^ As information carrier, DNA contains the individual canonical nucleotides in strands of defined sequences. This geometric arrangement drastically alters the photophysics. In particular, the interaction between adjacent nucleotides opens alternative reaction channels such as the formation of di‐nucleotide lesions or of UV‐induced charge‐transfer states.^3^

Charge‐transfer states are highly reactive and can, on the one hand, cause DNA damage.^4^ On the other hand, charge‐transfer states have been shown to promote the repair of DNA lesions.^5^ The repair activity strongly depends on the sequence where in particular dG‐containing sections are a key factor to self‐repair, as dG acts as a strong electron donor.^6^ Recently it has been shown that the cyclobutane thymine‐dimer lesion (T=T) in the sequence d(GAT=T) is strongly repaired under UV irradiation. This repair is explained by a charge transfer from the transiently formed charge‐transfer state d(G^.+^A^.−^).5a

In this study, we investigated the photophysical properties of dG‐containing short oligonucleotides. We applied transient UV‐pump/mid‐IR probe techniques to characterize short‐lived intermediate states. We identified transient charge‐transfer states through their vibrational modes and determined lifetimes and population as a function of sequence and oligonucleotide length. The experimental results are discussed in the context of DNA‐photoactivity.

## Results and Discussion

**Figure 1:**
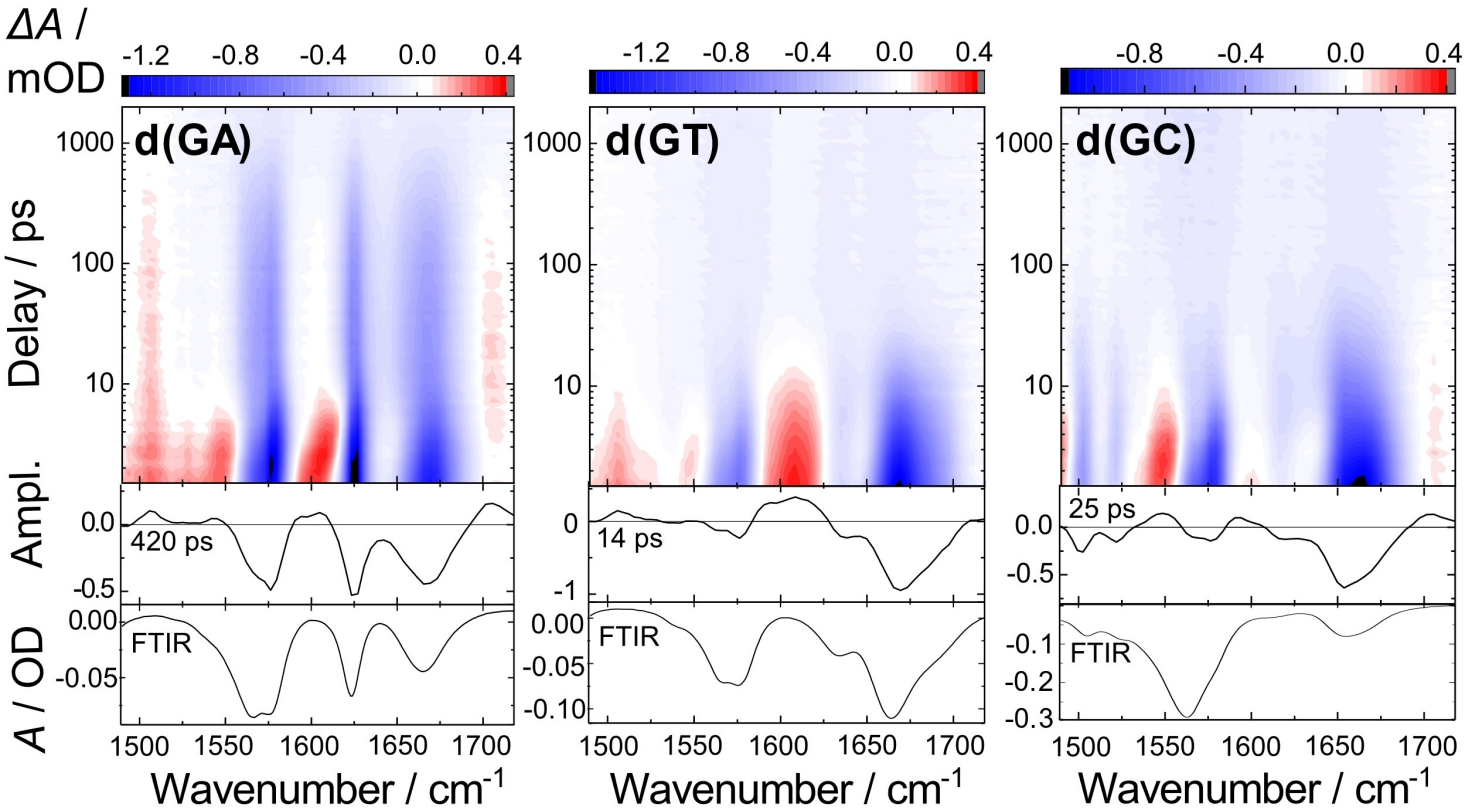
Transient IR absorption difference spectra of three dG‐containing dinucleotides after excitation at 266 nm. Top: Absorption changes plotted in contour representation (red: positive, blue: negative absorption changes). Bottom: Fitted decay‐associated difference spectra (DADS) corresponding to intermediate states with the indicated time constants. The inverted ground state spectra (FTIR) are shown for comparison.

In a first set of experiments, we investigated the dinucleotides d(GA), d(GT) and d(GC) by using transient UV‐pump and mid‐IR probe spectroscopy (Figure 1). The upper part shows the transient absorption difference spectra Δ*A* (color coded) plotted on a logarithmic timescale. Negative spectral bands (blue) are due to depopulation of the initial ground state and occur at wavenumbers corresponding to the bands found in the FTIR absorption spectra (Figure 1, bottom). Positive bands (red) are due to induced absorption, characteristic for excited states and photoproducts and are used, to identify the nature of the intermediates. At early times, within the first 10 ps, the absorption dynamics are dominated by excited state relaxation and vibrational cooling. Subsequent absorption dynamics can be assigned to charge‐transfer states and are discussed here in detail. Typically, the data can be modeled by a global fit with two time constants *τ*_1_ (in the 5 ps range, relaxation of excitation and vibrational cooling) and *τ*_2_ (related to the slower charge transfer dynamics). The time constants for all investigated samples are given in Table 1. The fit amplitudes, that is, the decay‐associated difference spectra (DADS) related to *τ*_2_ allow the spectroscopic characterization of the corresponding intermediate state (Figure 1).

**Table 1:** Excited‐state lifetimes and quantum yields of the charge separated states. All data are fitted with a multi‐exponential decay model, including 3 time constants for GC (*τ*_0_=1.1±0.4 ps) and 2 time constants for all other samples, following a global fitting routine.^14^ The quantum yields are estimated from the amplitudes of the absorption bleach in the DADS spectra (for details see the Supporting Information).

Sample	*τ*_1_/[ps]	*τ*_2_/[ps]	φ_CT_/[%]
d(GA)	4±1	420±120	42±20
d(GT)	5±3	14±5	66±35*
d(GC)	4±1	25±9	54±30*
d(AG)	4±1	280±160	32±15
d(GAAG)	5±2	500±180	56±30
d(GAGA)	5±2	630±200	55±30
d(GA)_10_	11±4	500±140	63±30

* For GT and GC the short decay time *τ*_2_ imposes additional difficulties for a quantitative determination of the quantum yields φ_CT_. Here the yields should be taken only as a rough estimate.

The data for d(GA) are presented in the left part of Figure 1. The spectral transients can be fitted by exponential functions with time constants of *τ*_1_=4±1 ps and *τ*_2_=420±120 ps. The three negative bleaching bands at 1576, 1627 and 1673 cm^−1^ are striking. The wave numbers correspond to the positions in the stationary FTIR absorption spectrum of d(GA) (Figure 1 bottom left). The two bands at 1576 and 1673 cm^−1^ are due to the bleaching of guanine, and the band at 1627 cm^−1^ is due to the bleaching of adenine absorption after excitation.^7^ The features found at early delay times (< ca. 5 ps)

correspond to absorption changes expected from the cooling of a vibrationally hot ground‐state.3c, 8 The final recovery of the ground state absorption occurs on the 400 ps timescale. The DADS of the long‐lived *τ*_2_=420 ps component is plotted in Figure 1 left, center. Here the positive peaks at 1608 cm^−1^ and 1704 cm^−1^ are due to the absorption of the guanine radical cation.3c, 9 The weak bands around 1510 and 1543 cm^−1^ correspond to an adenine radical anion band (calculated spectra in Figure S2). In agreement with previous studies we can assign the long‐lived component to the charge‐transfer state d(G^.+^A^.−^).3c

Results from the dinucleotide d(AG) are shown in Figure S1. They agree well with those obtained for d(GA). The marker bands in the DADS are the same. The observed time constants are *τ*_1_=4±1 ps and *τ*_2_=280±160 ps. The somewhat shorter time constant *τ*_2_ is in agreement with previous studies in the UV and visible range.2c, 3b, 10

The d(GG) dinucleotide is not treated in details since at a concentration of 5 mM, required for transient IR measurements, the UV/Vis spectrum deviates from the spectrum at low concentrations (Figure S4).^11^

**Figure 2:**
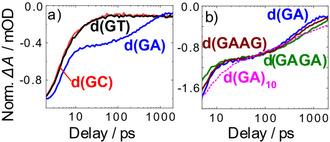
Time dependence of the IR absorption changes at the guanine ground‐state band around 1576 cm^−1^ after excitation at 266 nm. a) dG‐containing dinucleotides (normalized at the local minimum around 1.5 ps). b) dG‐ and dA‐containing oligonucleotides (normalized at a delay time t=51 ps).

In contrast to the pure purine dinucleotides d(GA) and d(AG) the pyrimidine containing dinucleotides d(GT) and d(GC) display a much faster recovery of the ground state (Figure 1). The data can be fitted again with a sum of exponentials d(GT): *τ*_1_=5±3, *τ*_2_=14±5 ps, d(GC): *τ*_0_=1.1±0.4 ps, *τ*_1_=4±1 ps, *τ*_2_=25±9 ps. The transient spectral changes on early times can again be assigned to vibrational cooling. However, the time constant *τ*_2_ of the slower process is found to be much shorter for the pyrimidine containing dinucleotides. This is evident when the temporal evolution of the ground state bleach at the guanine band (at 1576 cm^−1^) is plotted in Figure 2a for d(GT), d(GC) and d(GA). In the pyrimidine containing dimers the decay *τ*_2_ is accelerated by a factor of about 10. The DADS for d(GT) and d(GC) (central part of Figure 1) clearly show that the process *τ*_2_ not only represents the recovery of the ground state bands but also exhibits the characteristic positive marker bands for charge‐transfer states. For d(GC) positive amplitudes are found at 1704, 1592 (dG^.+^) and 1549 cm^−1^ (dC^.−^).3c The lifetime of the charge‐transfer state of d(GC) also agrees with d(CG) reported in a study by Takaya and coworkers.3b For d(GT) the guanine cation absorption at 1608 cm^−1^ is clearly seen while a shoulder appears at >1704 cm^−1^.

The broad positive band in the DADS of d(GT) around 1608 cm^−1^ may be caused by the overlap of the bands of the dT^.−^ anion and the dG^.+^ cation.3d The corresponding calculated spectra are shown in Figure S2. The comparison of measured absorption changes (DADS) with the calculated band positions (see the Supporting Information) clearly shows, that the longer‐lived components in all studied dinucleotides are radical pair states where the electron is transferred from the electron donor dG to the acceptor dA, dT or dC. The radical pair state has a lifetime in the picosecond range (15 to 600 ps). The efficiency of this charge transfer reaction can be estimated from the amplitudes of the absorption bleach in the DADS spectra (for details see the Supporting Information). The efficiency of charge transfer (*φ*_CT_) amounts to around 50 %. Details are given in Table 1 for the different oligonucleotides. Although charge‐transfer states are formed in all dinucleotides with similar amplitudes, there is a strong acceleration of the charge recombination in the pyrimidine containing dinucleotides. This effect can be described by Marcus theory.3b, 12 The charge recombination typically takes place in the Marcus inverted regime, where a lower thermodynamic driving force is related with a faster charge recombination. Since the driving force, related to the redox potential difference is lower for the purine‐pyrimidine than for the purine‐purine charge recombination,6a, 12 the lifetimes *τ*_2_ should be shorter for d(GC), d(GT), which is observed experimentally (Table 1).

**Figure 3:**
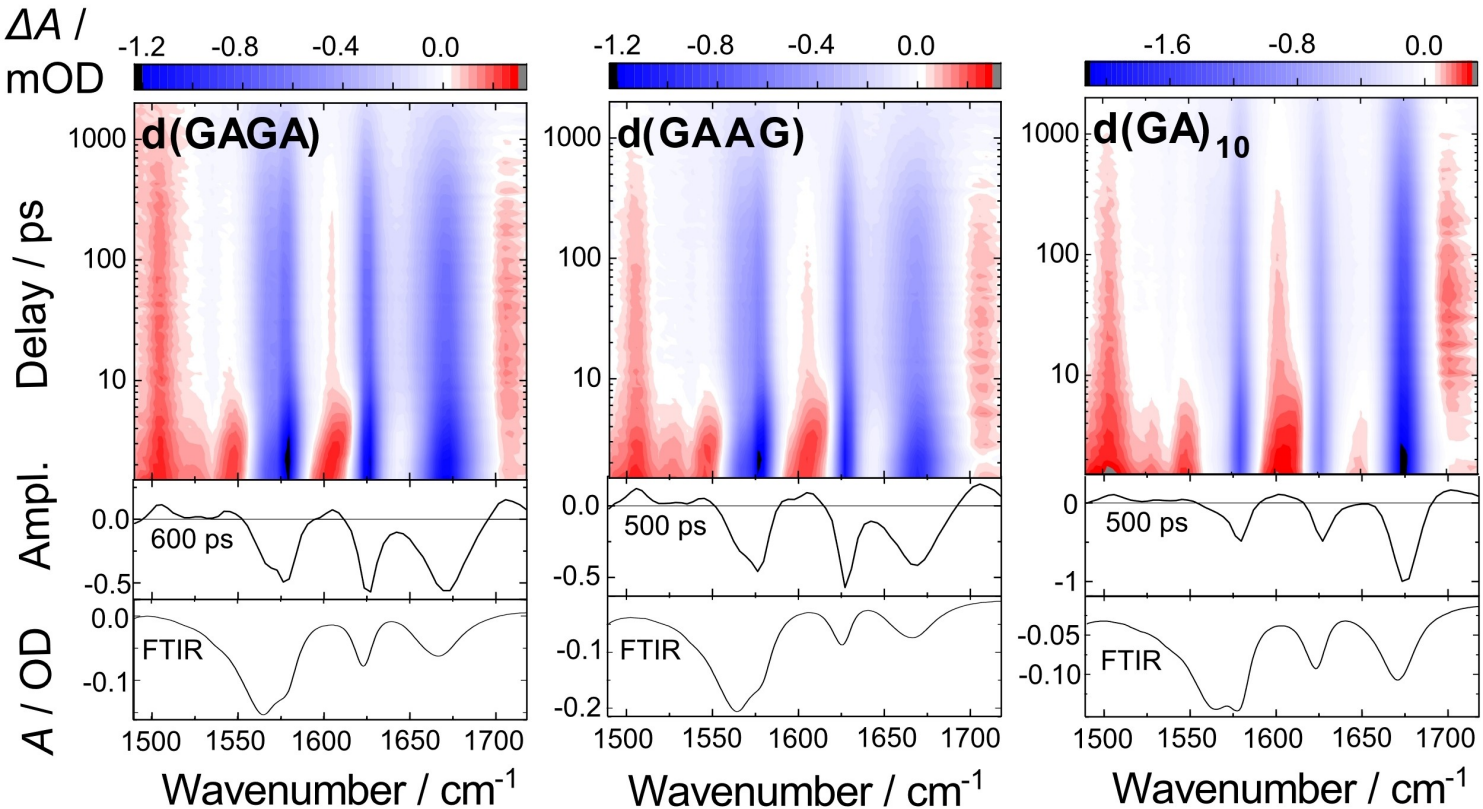
Transient IR absorption difference spectra of three dG‐ and dA‐containing oligonucleotides after excitation at 266 nm. Top: Experimental data in contour representation (red: positive, blue: negative absorption changes). Bottom: Fitted decay associated difference spectra (DADS) corresponding to intermediate states with the indicated time constants. The inverted ground state spectrum (FTIR) is shown for comparison.

In a second set of experiments, we address the influence of oligonucleotide length on the formation and lifetime of charge‐separated states. For this purpose, we choose the two tetranucleotides d(GAGA), d(GAAG) and the alternating 20‐mer sequence d(GA)_10_ as samples (Figure 3). The over‐all spectral and temporal features resemble those of the d(GA) dimer: The band positions are very similar. The absorption transients at later times also involve biexponential kinetics with the slow components in the 500 ps range. The corresponding absorption features are again indicative for the presence of radical pair states. Figure 2b shows the normalized absorption transients at the ground state absorption band of dG (ca. 1576 cm^−1^) for the different d(GA)‐containing oligonucleotides. In this plot, the qualitative similarity of the transients becomes apparent. In details the curves show some weak differences, pointing to different decay times (results given in Table 1). For d(GA)_10_ the observed differences can be assigned to structural inhomogeneities.^13^ The data on the oligonucleotides d(GAGA), d(GAAG), and d(GA)_10_ show a weak trend towards longer lifetimes *τ*_2_ of the radical pair states as compared to those of the dinucleotides d(GA) and d(AG). For d(GAGA) we find 630±200 ps, for d(GAAG) 500±180 ps and for d(GA)_10_ 500±140 ps.

## Conclusions

Time‐resolved IR spectroscopy was used to study photophysical dynamics of dG‐containing di‐ and oligonucleotides. After excitation in the UV at 266 nm we found transient absorption changes pointing to excitation decay and vibrational cooling on the 5 ps timescale. Additional slower decaying intermediates were observed in all samples and assigned to charge‐transfer states by comparison with the literature and by density functional calculations. While the lifetimes of the charge‐transfer states of d(GT) and d(GC) were around 20 ps, the pure purine containing oligonucleotides showed lifetimes in the 300 to 600 ps range. We found yields of the charge‐transfer states in the range of 50 %. The observations clearly show that transient charge‐transfer states are efficiently formed in dG‐containing oligomers. The known reactivity and the high formation yield make charge‐transfer states important promoters for secondary reactions in DNA or in its direct surroundings.

## Experimental Section

The oligonucleotides were purchased from biomers.net (Ulm, Germany). The lyophilized and HPLC purified samples were dissolved in 50 mM phosphate buffer (KH_2_PO_4_, Na_2_HPO_4_) in D_2_O (Sigma Aldrich). The oligo concentrations were kept at ∼10 mM per base. The pump probe experiments were performed in a flow cell with BaF_2_ windows (sample thickness 100 μm) under ambient oxygen conditions at a temperature of about 23 °C.

The basic principles of the pump probe setup for time‐resolved experiments has been described previously.^15^ The light source in the pump and probe setup was a Ti : Sa based laser amplifier system (Libra, Coherent) with an output pulse duration of ∼113 fs at 800 nm and a repetition rate of 1 kHz. The third harmonic (266 nm) of the fundamental was used for the excitation (pump).15c The excitation energy at the sample position was 2.2 μJ with a spot diameter of 150 μm FWHM. The pump pulse was transmitted through a fused silica rod of 25 cm length to increase its pulse duration to about 1 ps. The broadband mid‐IR light between 5–7 μm (probe) was generated by a cascade of two optical parametric amplifiers (OPA) producing signal and idler pulses at 1400 and 1800 nm and difference frequency mixing in a silver thiogallate crystal (AgGaS_2_). Pump and probe pulses were focused and spatially overlapped in the sample. After passing the sample the probe light was spectrally dispersed (Chromex 250IS, Bruker) and detected on a 64‐channel MCT array (IR‐0144, Infrared Systems Development). All experiments were performed under magic angle conditions. The analysis of the transient data uses global fitting where the uncertainty of the time‐constants was determined by an exhaustive search procedure. The transient and stationary IR spectroscopy and the density functional theory calculations follow the procedures described by Bucher et al.3c, 16

## Conflict of interest

The authors declare no conflict of interest.

## Supporting information

As a service to our authors and readers, this journal provides supporting information supplied by the authors. Such materials are peer reviewed and may be re‐organized for online delivery, but are not copy‐edited or typeset. Technical support issues arising from supporting information (other than missing files) should be addressed to the authors.

Supplementary
