# Supplementary material for: UV‐Induced Charge‐Transfer States in Short Guanosine‐Containing DNA Oligonucleotides
Source: Chembiochem. 2020 May 5;21(16):2306–10. doi: 10.1002/cbic.202000103 (PMC7496882; doi:10.1002/cbic.202000103)
Supplement: Supplementary file 1 — Supplementary [file CBIC-21-2306-s001.pdf]

ChemBioChem

Supporting Information

UV-Induced Charge-Transfer States in Short Guanosine-Containing DNA Oligonucleotides

Corinna L. Kufner, Wolfgang Zinth, and Dominik B. Bucher*© 2020 The Authors. Published by Wiley-VCH Verlag GmbH & Co. KGaA. This is an open access article under the terms of the Creative Commons Attribution License, which permits use, distribution and reproduction in any medium, provided the original work is properly cited.

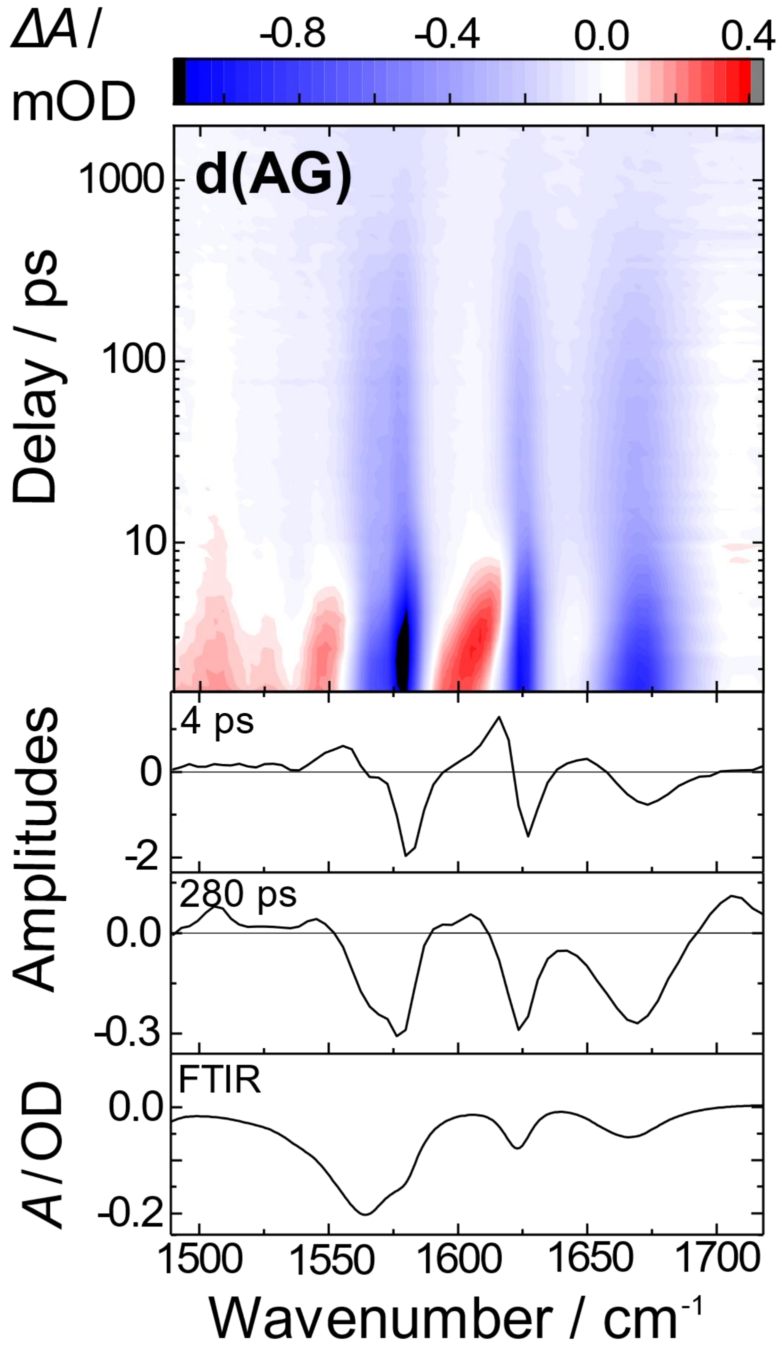

Figure S1: *Transient IR absorption difference spectra of the dinucleotide d(AG) after excitation at 266 nm. Top: Experimental data in contour representation (red: positive / blue: negative absorption changes). Middle: Fitted decay associated difference spectra (DADS) corresponding to intermediate states at the given time constants. The positions of the radical ion marker bands around 1550 cm^{-1}, 1608 cm^{-1} and 1704 cm^{-1} agree with previously published data.[1] The inverted ground state spectrum (FTIR) is shown for comparison. For calculated spectra of dG$^{•+}$ and dA$^{•-}$ see Figure S2.*

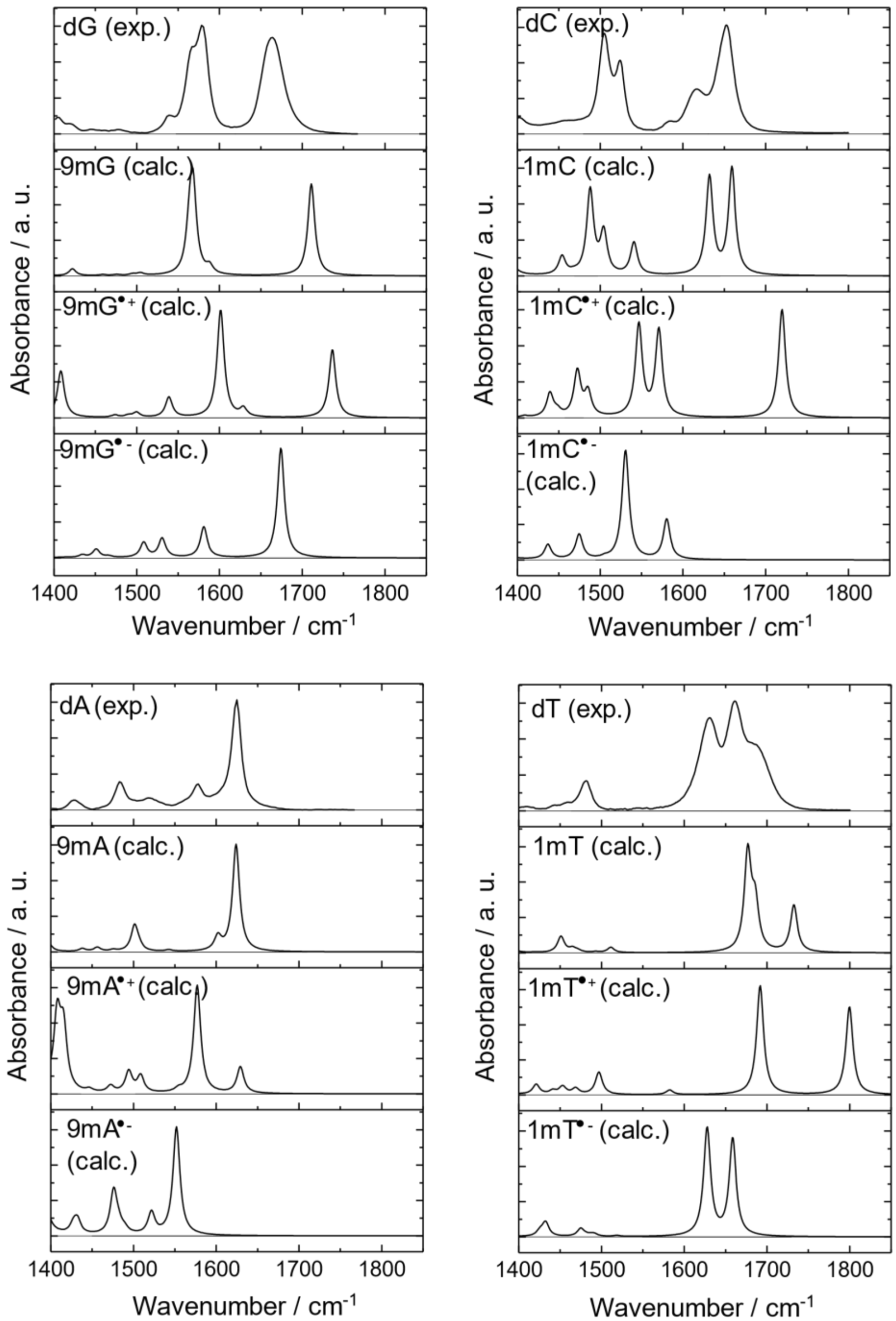

***Figure S2**: Experimental absorbance spectra (adapted from [2]) of the canonical DNA nucleosides in comparison with calculated absorbance spectra of the ground states and radical ions.[1] For convenience the sugar was substitutes by a methyl-group in the calculations.[3]*

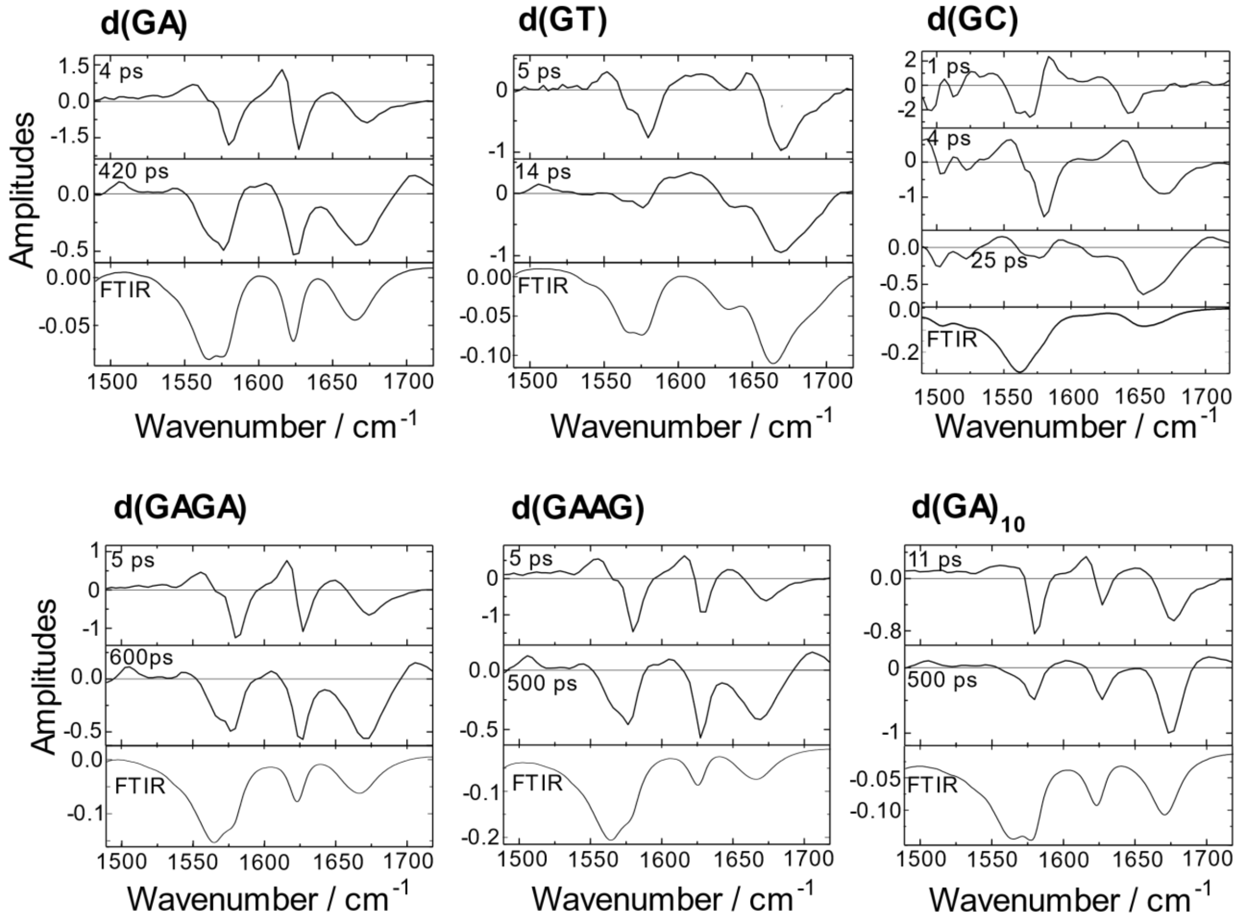

Figure S3*: Comparison of decay associated difference spectra and FTIR spectra for the investigated samples. Top: Fitted decay associated difference spectra (DADS) corresponding to intermediate states at the indicated time constants (black) following excitation at 266 nm. The inverted ground state spectra (FTIR) are shown for comparison. For calculated radical ion spectra see figure S2.*

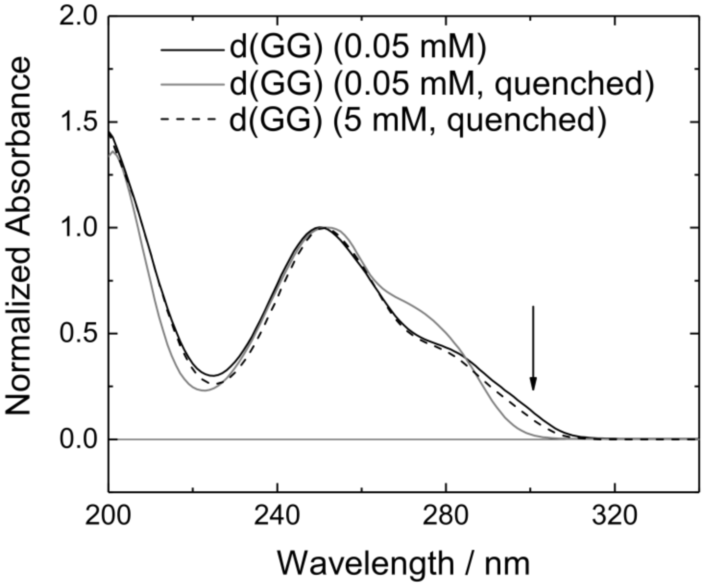

***Figure S4**: Normalized UV / Vis spectrum of the d(GG) dinucleotide at a concentration of 0.05 mM in buffered D_2O solution (black). The gray curve shows a spectrum of the same sample heated to 86°C for 3 min followed by quenching on ice (gray) in order to disintegrate molecular aggregates. The absorbance changes around 290 nm (arrow) point to an aggregation of the sample.[4] At high concentrations of 5 mM, which are required for transient IR absorption experiments, the thermal quenching did not affect the spectral shape significantly (black dashed). Apparently, the procedure did not reduce the aggregation. Therefore, the d(GG) dinucleotide sample was not treated in this publication.*

Density Functional Theory Calculations. The harmonic vibrational frequencies were calculated with the Gaussian 03 software using Becke3Lyp 6-311+G** Opt Freq = Read Isotopes PCM Solvent = Water functional with deuterated amino and imino side groups.[1, 3] All calculations were performed for the 1-(pyrimidines) or 9- (purines) methyl-subsitituted nucleobases for simplicity. For each molecule the geometry was optimized prior to the vibrational frequency analysis.

Efficiency of the Charge Transfer States. The efficiency of the charge transfer reactions for each sample is estimated from the absorption changes recorded in the strong ground state bands at ~1670 cm^{-1} and / or ~1627 cm^{-1}. At these positions one expects no absorption from radicals. Thus, the decay of the charge transfer state leads to a recovery of the ground state absorption. The corresponding absorption change (recorded as the decay associated difference spectrum, DADS) is proportional to the number N_{CT} of molecules decaying from the charge transfer state to the groundstate, $\Delta A_{DADS,\tau 2} = N_{CT}\,\sigma_i$ (σ_i is the absorption cross-section at the observation wavenumber). The amount of excitation, i. e. the number of absorbed photons $N_{h\nu}$ is needed to calculate the quantum efficiency $\varphi_{CT} = N_{CT} / N_{h\nu}$. $N_{h\nu}$ is determined by measuring the absorption decrease ΔA_{bleach} found at the peak of the bleaching (close to t = 2 ps). At this time the excitation pulse is finished and only a small fraction of the molecules in the charge transfer state has started to decay. Thus the originally excited molecules are either in the electronically excited state, in the electronic groundstate with high vibrational excess energy, or in the charge transfer state.

In these states the original groundstate absorption has vanished (see e.g. [5]). Under these conditions the absorption change ΔA_{bleach} amounts to $\Delta A_{bleach} = N_{h\nu}\, \sigma_i$. The quantum yield can now be calculated as $\varphi_{CT} = N_{CT} / N_{h\nu} = A_{DADS} / \Delta A_{bleach}$. The error of the quantum yield given in Table 1 considers statistical variations, the uncertainty in the determination of the DADS, a possible decay of the charge transfer state within the first 2 ps and the potential interference due to absorption from adjacent bands.

[1] D. B. Bucher, B. M. Pilles, T. Carell, W. Zinth, *Proc. Natl. Acad. Sci. U. S. A.* **2014**, *111*, 4369-4374.

[2] W. J. Schreier, P. Gilch, W. Zinth, *Annual review of physical chemistry* **2015**, *66*, 497-519.

[3] M. J. Frisch, G. W. Trucks, H. B. Schlegel, G. E. Scuseria, R. M. A., C. J. R., J. Montgomery, J. A., T. Vreven, K. N. Kudin, J. C. Burant, J. M. Millam, S. S. Iyengar, J. Tomasi, V. Barone, B. Mennucci, M. Cossi, G. Scalmani, N. Rega, G. A. Petersson, H. Nakatsuji, M. Hada, M. Ehara, K. Toyota, R. Fukuda, J. Hasegawa, M. Ishida, T. Nakajima, Y. Honda, O. Kitao, H. Nakai, M. Klene, X. Li, J. E. Knox, H. P. Hratchian, J. B. Cross, V. Bakken, C. Adamo, J. Jaramillo, R. Gomperts, R. E. Stratmann, O. Yazyev, A. J. Austin, R. Cammi, C. Pomelli, J. W. Ochterski, P. Y. Ayala, K. Morokuma, G. A. Voth, P. Salvador, J. J. Dannenberg, V. G. Zakrzewski, S. Dapprich, A. D. Daniels, M. C. Strain, O. Farkas, D. K. Malick, A. D. Rabuck, K. Raghavachari, J. B. Foresman, J. V. Ortiz, Q. Cui, A. G. Baboul, S. Clifford, J. Cioslowski, B. B. Stefanov, G. Liu, A. Liashenko, P. Piskorz, I. Komaromi, R. L. Martin, D. J. Fox, T. Keith, M. A. Al-Laham, C. Y. Peng, A. Nanayakkara, M. Challacombe, P. M. W. Gill, B. Johnson, W. Chen, M. W. Wong, C. Gonzalez, J. A. Pople, Gaussian Inc., Wallingford, **2004**.

[4] a) L. Martínez-Fernández, A. Banyasz, D. Markovitsi, R. Improta, *Chemistry–A European Journal* **2018**, *24*, 15185-15189; b) P. Changenet-Barret, Y. Hua, D. Markovitsi, in *Photoinduced Phenomena in Nucleic Acids II*, Springer, **2014**, pp. 183-201; c) F.-A. Miannay, A. Banyasz, T. Gustavsson, D. Markovitsi, *The Journal of Physical Chemistry C* **2009**, *113*, 11760-11765.

[5] P. Hamm, S. Ohline, W. Zinth, *The Journal of chemical physics* **1997**, *106*, 519-529.
